# Research trends and network approach of critical thinking skills in English Language Teaching – A bibliometric analysis implementing R studio

**DOI:** 10.1016/j.heliyon.2025.e42080

**Published:** 2025-01-17

**Authors:** M.P. Arthi, S.N.S. Gandhimathi

**Affiliations:** Department of English, School of Social Sciences and Languages, Vellore Institute of Technology, Vellore, India

**Keywords:** Critical thinking, English language teaching, Bibliometric analysis, R studio, Biblioshiny

## Abstract

Critical thinking is often regarded as one of the vital skills in the 21st century. In recent years, it has become important to combine this skill with other skills such as language learning, creativity, problem-solving, decision making, and reflective thinking. As language and thoughts are intertwined and influence each other, incorporating thinking skills into English Language Teaching (ELT) can enhance academic and professional success. The prime objective of the current study is to conduct a bibliometric analysis of research on Critical Thinking (CT) in ELT from 2012 to 2022 by focusing on publication and research trends such as influential authors, globally cited documents, pertinent journals, leading countries, and significant affiliations. The current study also sheds light on the co-occurrence of keywords and thematic evolution through bibliometric analysis, examining 238 articles from Scopus and Web of Science databases using R studio (Biblioshiny). This bibliometric analysis highlights *Thinking Skills and Creativity* as the most pertinent journal with high citation count and China being the most cited country in this field. It also accentuates the interconnection between higher-order thinking skills, self-regulation, critical thinking, and critical reading. The thematic evolution of CT shows a shift from collaborative learning and CT (2012–2019) to language teaching and CT (2020–2022). It emphasizes the importance and the rising prominence of CT skills in language teaching. Moreover, this review will guide scholars and academicians to identify future research directions in the field of CT.

## 1 Introduction

The integration of Critical Thinking (CT) skills with domains such as philosophy, cognitive psychology, linguistics, and language arts confers different perspectives on thinking process. Language arts such as English Language Teaching (ELT) and oral communication address the necessity of incorporating CT skills into the curriculum. Thinking is structured and influenced by language since the thoughts, utterances, emotions, and experiences of humans are conveyed through language. Appropriate use of language serves as a means to elucidate the cognitive process and it is crucial to employ precise and relevant language, as it results in lucid and accurate thinking. Considering the intimacy between language and thought, language proficiency is a prerequisite for expert thinkers. As language mimics thinking, darning language with critical thoughts improves CT abilities [1]. In the 21st century, CT has become one of the most essential abilities, making it crucial for students to develop this skill. The importance of instilling CT in learners is growing every day as it significantly influences their linguistic abilities [2]. The wide-ranging attention on CT skills has been highlighted in recent research, implying an inextricable link between cognition and linguistic development. Learners are encouraged to acquire the English language because of its remarkable growth and the inevitability of language skills in a futuristic working environment. Incorporating CT into English language instruction enables students to develop language proficiency while using it as a medium to critically examine diverse topics. However, the establishment of CT in ELT is not prevalent, as emphasis has been placed solely on learners' language abilities. Therefore, the investigation of CT skills related to second language acquisition has received less attention, which could be attributed to teachers’ uncertainty about the integration of CT skills in ELT. Teachers should promote positive attitudes toward CT skills as they take on a supportive role. Besides, students need to become aware of their cognitive processes to enhance their CT abilities, which will ultimately lead to significant improvements in their language proficiency [3,4].

Critical thinking is the ability to analyse and examine ideas. An individual who thinks critically grasps and comprehends abstract concepts effectively in a given context. Integrating CT into ELT benefits the learners by engaging them to discern contexts and concepts. CT also necessitates effective communication, thereby fostering listening, speaking, reading, and writing skills. As CT merges well within every discipline, it is indispensable for learners of all ages and disciplines [5].

Language acts as a fundamental thinking instrument as it has its effect on the cognitive process. In today's job market, the importance of CT is evident, as employers globally seek candidates with substantial CT and language proficiencies. This underscores the complex interplay between these skills in the current era [6]. Language plays a generative role in stimulating the thought process and it is indistinguishably linked to the association between mind and thought [7]. The ability to communicate effectively in a language is feasible only if the learners are able to think critically. The prevalence of CT in education has become crucial as there is a need to teach English language skills with CT competencies. Considering the criteria for a global workspace, there is a dire need to blend these skills into the curriculum at every level of education [8]. The relevance of the English language in the global market of the 21st century is highlighted where it suggests that the implementation of instructional techniques, such as triggering, exposing, guiding, and extending will benefit both teachers and learners involved in CT [9].

The national policy documents of 152 countries prioritized inculcating skills, such as communication, CT, creativity, and problem-solving skills, in their teaching strategies among the learners. Among all other skills, namely information technology, social, and entrepreneurship skills, CT holds fourth place as the recurrently identified skill in national policy documents, which states its relevance in the current global competitive world. Introducing twenty-first-century skills into academic disciplines, such as Language, Mathematics, Natural Sciences, and History, is also gaining prominence. Curriculum reforms in countries such as the Philippines, Australia, and Kenya have altered educational goals by implementing a unique pedagogical strategy that stresses on the development of CT. For instance, a high school in Melbourne has shifted its educational approach by emphasizing critical and creative thinking in its curriculum [10]. The ‘making thinking visible’ is a vital approach to be implemented in the classroom to enhance the understanding and knowledge of the learners. By integrating thinking into the classroom, learners have the opportunity to explore the complex relationship between language and thought. This approach equips learners for real-world problems and supports lifelong learning. Therefore, it plays a critical role in developing learners' CT skills and fostering the capacity to apply the old concepts to new situations. The intricate connection between language and cognition is indispensable, as it recognizes language as a means of conveying thoughts and also as a catalyst for the development of thinking [3,11].

### 1.1 Rationale and significance of the research

Critical thinking has become an essential competency in the global education system. It reflects its relevance in the National Educational Policies (NEP) and curricula of several countries. For instance, NEP of Japan has emphasized the importance of CT skills for fostering innovation and problem-solving among the students [12]. Similarly, Singapore has introduced the 'Skills Future' policy to incorporate CT into lifelong learning. This policy highlighted the value of CT in developing a resilient and adaptable workforce [13,14]. In the United Kingdom, CT is acknowledged as a vital skill for academic and professional proficiency [15,16]. Furthermore, the effect of AI on employment opportunities has emphasized the importance of CT in the current era, as it is a key objective of educational institutions and is being incorporated into higher education curricula throughout Europe [17,18]. The NEP of India stressed the necessity of cultivating CT and envisioned a transformation in the education system that fosters a learner-centric approach [19]. Thus, it is explicit that CT is recognized globally, in the academic programs of various countries. Considering its significance in global educational policies, it has been observed that there is a dire need to carry out a comprehensive bibliometric analysis. It is also noted that a bibliometric review plays an important role in identifying the prevailing trends and gaps in the literature on CT, especially in the field of ELT. It may enable the policy makers and academicians to adapt their teaching strategies to emerging research findings.

In order to systematically understand the evolving landscape of CT in ELT, the present study carried out a bibliometric analysis to map and track scientific advancements in this field. This enables researchers and educators to attain “a one-stop overview” of the field and identify “novel areas of investigation.” In addition, it provides a detailed exploration of several critical aspects, such as annual scientific production, prominent journals, average article citations, globally cited research work, important authors, relevant affiliations, influential countries, tree maps, and network visualization of high-frequency keywords. These aspects provide valuable insights into the intellectual growth of this research field. Publication trends are crucial for researchers and policymakers to recognize their evolution over time. In addition, identifying the most prominent journals related to this field allows researchers to understand where the highest-impact studies are being disseminated and to serve as platforms for future research contributions.

The average number of citations per article is a key metric for evaluating the impact of research and the globally cited research works demonstrate the significance of foundational and cross-disciplinary studies. These highly cited works not only influence the field of language teaching but also have wide-ranging implications in other domains. Policymakers often rely on groundbreaking studies to shape their curriculum development and teaching methodologies. Furthermore, the most cited authors enable scholars to engage with the most impactful research conducted by leading experts and the authors’ affiliations reveal the institutions that play a pivotal role in conducting research on CT and ELT. This insight is indispensable for researchers seeking collaboration and policymakers interested in exploring the hubs of educational innovation. Additionally, distinguishing the most-cited countries highlights their global influence, as they are always at the forefront of implementing educational reforms that emphasize CT skills. Moreover, the treemap and network visualization of keywords reveal the dominant themes. It visualizes how these terms are interconnected and acts as a tool to uncover deep insights into the structure, evolution, and future directions of the research area [20]. Therefore, it is necessary to review these aspects to shed light on the evolution of research on CT related to ELT and to guide future studies, academic collaboration, and policy formation in this field.

### 1.2 Research questions (RQ)

Curating a bibliometric summary of CT in the field of ELT will reveal extensive research aspects in this area. This review is of great significance as it aims to (1) analyse the trends in publications related to the development of CT skills in ELT, (2) identify influential authors, globally cited documents, and journals, (3) determine the top cited countries and significant affiliations connected to this study and (4) explore the research trends and thematic evolution in CT research through network approach. As a result, this review article will facilitate further research in the field of CT in ELT by enlightening the research scholars and educators all over the world.

• RQ1: What is the annual scientific production and growth rate of CT research in ELT?
• RQ2: Who or what are the most relevant journals, authors, and globally cited documents?
• RQ3: What are the most pertinent affiliations and countries involved in CT research on ELT?
• RQ4: What are the evolving trends, top keywords, keyword co-occurrence, and thematic evolution of CT skills in ELT over time?

Research questions are essential to carry out bibliometric analysis as they play a crucial role in enhancing the overall rigor and relevance of CT research in ELT. Especially, RQ1 examines trends in publication by focusing on the annual increase in research articles and the growth rate of CT in ELT. RQ2 assesses the relevance and impact of journals, authors, and globally cited documents in the field. RQ3 explores the most cited countries and affiliations in the field. The objective of RQ4 is to uncover emerging research trends, including prominent keywords, keyword co-occurrences, and the thematic development of CT skills in ELT. These research questions will enable scholars and educators to identify areas of prominence and potential gaps in the literature. This confirms that the bibliometric analysis is pertinent to the academic community by resolving specific knowledge gaps or issues of interest, thereby promoting advancements in the field of CT in language teaching. Thus, the research questions mentioned above make the current study more profound and applicable.

## 2 Review of literature

### 2.1 Overview of previous studies on bibliometric analysis

R-tool is flexible in nature and it revealed the perpetuity and adaptability of bibliometrics. A replicable review process can be implemented using bibliometrics, since it is highly objective in nature and involves a wide range of analytics and software. Therefore, bibliometrics ensures objectivity and entails numerous statistical and graphical techniques [21]. A bibliometric analysis of previous studies on teachers' CT using R studio (Biblioshiny) underscores the most relevant sources and research hotspots, indicating future directions for research in this area. It illustrated the hotspots of a particular research area and explored the productivity of research on teachers’ CT skills [22]. Thus, the bibliometric analysis has garnered tremendous popularity in recent years and is a scrupulous method for examining scientific data. The bibliometric methodology offers a refined overview of the field, enabling researchers to identify gaps in a research area through intellectual structure and thematic evolution [20].

Bibliometrics involves identifying patterns in a specific area and it can be used to ensure research priorities. An analysis of 20,268 publications on bibliometrics from multiple databases revealed that there is a significant rise of popularity in this field recently. The study provided an overview of bibliometric research, tracing it back from the 12th century and involving citation indices from Hebrew to the modern usage of bibliometrics. The articles from leading journals were taken into consideration and bibliometric analysis was performed using R studio. The network of articles disclose how bibliometrics turned out to be a definite part of scientific research [23]. A recent study applied R studio to analyse the documents which are imported from the *Scopus* database. It used *Biblioshiny* to carry out data visualization and found the outcomes of the bibliometric analysis reliable and verifiable. These findings reveal developing tendencies in this particular research area. In addition, graphical visualization of the results illustrated the core elements of the analysis performed using R studio [24].

### 2.2 Emerging trends in ELT

An extensive bibliometric analysis of previous studies in language and linguistics among 13 Asian countries, revealing popular topics, such as language education, discourse analysis, and sentiment analysis was conducted [25]. Key research areas such as technology integration, language policy, and cultural orientation were identified through a bibliometric study from 2013 to 2022 which revealed the influential journals, authors, keywords, and themes in ELT research [26]. A similar bibliometric study highlighted the importance of ELT in shaping learners' intellectual development and communicative competence in contemporary education. This study sheds light on the evolution of education and the impact of emerging pedagogical strategies in ELT [27]. Recent studies have highlighted the integration of Artificial Intelligence (AI) into ELT and they have used CiteSpace visualization software to analyse research trends in ELT, particularly highlighting the growing importance of AI in language teaching. This demonstrated the increasing attention given to AI in education, specifically in the field of ELT and explored the research hotspots, including educational theory, information technology, and examples of AI in teaching and learning [28]. Similarly, a study examined factors such as cultural values, teacher influence, and the classroom environment that influence learners’ willingness to communicate in a second language. This review offered a bibliometric analysis of research on Willingness to Communicate (WTC) in the context of English as a Second Language (ESL) [29]. The effect of COVID-19 on language teaching and learning highlighted the need for ongoing professional development, such as revised teacher education programs, updated assessment guidelines, and improved teacher-student digital literacy skills to shape the future of language education in the post-pandemic era [30].

Teachers are enhancing their skills and engaging in reflective practices to keep pace with innovations in ELT to equip students with necessary skills for effective communication in a globalized world, which necessitates adaptation to evolving methodologies and integration of emerging technologies in language education. The current trends in ELT include using technology for interactive learning experiences, emphasizing communicative and cultural proficiency, adopting task-based learning and inclusive strategies, and focusing on CT skills and personalized learning approaches [31]. A recent study underscores the transformative impact of technology on English language teaching and learning, reiterating the significance of integrating innovative tools and platforms to improve language acquisition and proficiency. Teachers in technology-enhanced classrooms play a vital role as facilitators, guiding students in using technology effectively for learning. They curate digital content, selecting high-quality resources that align with the curriculum and learning objectives [32].

A recent study conducted a comprehensive bibliometric and content analysis of AI integration in language learning. The researchers used tools like VOSviewer and R studio to identify influential authors, institutions, countries, and documents in the field. Their findings indicated that AI in language education shows significant potential for expansion and development [33]. The use of AI in English Language Teaching and Learning (ELT/L) has increased significantly, especially in higher education. This study also highlighted the challenges faced, such as technology breakdowns, limited capabilities of AI systems, and the fear of relying on technology. Additionally, it suggested that future research should focus on the impact of AI on the learners [34].

Crucial texts like Common European Framework of Reference for Languages (CEFR) and Essential Learnings (EL) are examined and suggested that there is a need for ELT policies to align with the actual use of English in international settings, advocating for a shift from native-speaker-focused methods to embrace the diverse role of English in this era [35]. The importance of incorporating Global Englishes in language teaching is emphasized and this study reviewed all the previous studies on linguistic diversity and innovative teaching practices. The major finding is that the inclusion of Global Englishes in higher education leads to a better understanding of linguistic and cultural diversity among the learners [36]. The role that the teachers play in adapting to new trends in language education is underscored, while another study explores emerging trends in Global English Language Teaching (GELT), such as postmethod pedagogy, digital technology use, intercultural communicative competence, critical pedagogy, teachers' professional development, localization of ELT, and learners’ autonomy [37,38].

## 3 Methodology

Bibliometrics is a quantitative method for analysing scientific data and it is an open-source science mapping statistical analysis tool written in *R* language to measure productivity within a research topic [21]. The methodology employed in the current study includes bibliometric analysis and the scientific data consist of documents exported from the Scopus and Web of Science databases. The analysis was carried out using the Preferred Reporting Items for Systematic Reviews and Meta-Analyses (PRISMA) 2020 checklist [65]. Furthermore, documents are obtained through a topic search on CT skills related to ELT. The database search strings included keywords such as ‘critical thinking skills’ OR ‘analytical skills’ AND ‘English Language Teaching’. Records that matched the search terms in the title, abstract, or keywords that focused on CT research in ELT were found. Data recovered from the Web of Science and Scopus databases on 1st January 2023, yielded 857 documents, which were analysed using R studio to mitigate duplicates found in both files, including the deduplication process, by eliminating duplicate documents from both files.

### 3.1 Data analysis

**Fig. 1:**
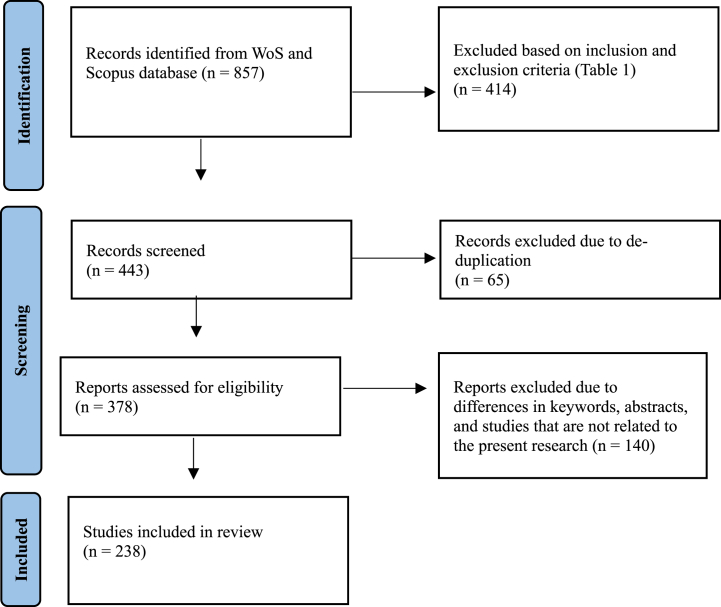
PRISMA flow diagram.

The inclusion and exclusion criteria mentioned above were applied to 857 articles, resulting in 443 articles as shown in Fig. 1. Among these, 65 duplicates were identified and the remaining 378 articles underwent manual screening in phase 2 based on their titles, keywords, and abstracts. During this screening process, all the titles, keywords, and abstracts of 378 articles were manually evaluated for eligibility to be included in the final review for bibliometric analysis. A total of 140 articles were deemed irrelevant to the context of the study was excluded. Some articles were excluded because they did not focus on CT skills, ELT, or second language learning. Instead, they focused on CT skills in the fields of science education and mathematics. After phase 2 screening, 238 articles were included in the bibliometric analysis. This software was used for data loading, conversion, and bibliometric analysis. Bibliometric analysis statistically assesses the academic quality of journals, revealing trending and emerging topics from 2012 to 2022.

The articles were analysed using R studio (Biblioshiny) for bibliometric analysis. It extracts and processes the complete bibliographic data from an Excel file uploaded in Biblioshiny. The purpose of this tool is to provide an overview of the data and insights into the current state and future direction of CT in ELT (see Table 1).

Table 2 provides the details of the data retrieved from the Scopus and Web of Science databases. It provides the timespan of the articles analysed through bibliometrics and the sources they are taken from, such as journals and the number of documents. The annual growth rate was 22.16 %, stating the continuum of research on CT skills in ELT. The content of the documents, including the keywords used by the authors, the percentage of international co-authorships, annual scientific production, globally cited documents, influential authors, affiliations and collective collaborations between countries were obtained through R studio (*Biblioshiny)*.

**Table 1:** Inclusion and exclusion criteria.

Criteria	Inclusion	Exclusion
Language	English	Other than English
Timeline	2012–2022	Earlier than 2012
Literature	Journal (research articles)	Other than research articles
Subject area	Arts and Humanities, Education Educational Research, Language Linguistics	Other than Arts and Humanities, Education Educational Research, Language Linguistics

**Table 2:** Data extracted from Scopus and Web of Science databases.

Metrics	
Timespan	2012:2022
Sources (Journals, Books, etc)	153
Documents	238
Annual Growth Rate %	22.16
Document Average Age	4.44
Average citations per doc	3.874
References	5110
Keywords Plus (ID)	184
Author's Keywords (DE)	782
Authors	562
Authors of single-authored docs	56
Single-authored docs	57
Co-Authors per Doc	2.45
International co-authorships %	7.143
Article	238

## 4 Results of the study

### 4.1 Publication trends

The extant research in ELT over the past decade has proved that CT is nurtured among the second language learners and it has been gaining attention worldwide. This is substantiated by the continuous scholarly output of articles projecting the advancement of CT skills within the purview of ELT, ranging from 2012 to 2022. The publication metrics underscore the central role that CT holds in the domain of Second Language Acquistion (SLA).

**Fig. 2:**
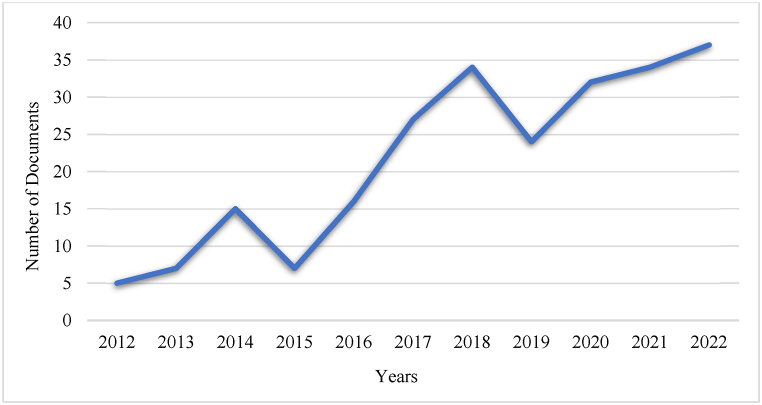
Annual scientific production of articles.

The perpetual nature of article production shows an increase in research output and it is evident from Fig. 2 that research on CT skills in ELT fluctuated from 2012 to 2018, with a steady increase in 2018. Research on CT was in the budding stage in 2012, whereas substantial development of articles was apparent until 2015. The upsurge in articles in this field was explicit in 2018 and the proliferation of articles from 2018 to 2022 demonstrates the growing importance of CT in ELT. Fig. 2 depicts the uniformity in the integration of CT skills in ELT; fostering CT has drawn the interest of researchers worldwide. As shown in Fig. 2 the number of scientific articles produced each year about CT abilities in ELT was quite low, whereas it gradually increased with more than 30 articles published in 2022 on the topic of CT in ELT. This shows the pivotal period of the advancement of CT research and exemplifies the pivotal juncture in this field from 2012 to 2022.

### 4.2 The local impact of prominent journals on CT in ELT

The local impact of influential journals in this field sheds light on the specific publication outlets that drive the research agenda and discourse germane to the development of CT skills in language education. To gain a deeper understanding of the research landscape in this area, it is crucial to examine the significance of the leading journals that publish research work on CT in ELT.

The ten most relevant journals in this field, based on their local impact, are listed in Table 3. *Thinking Skills and Creativity* is the most influential source, with 61 citations. This ensures that the journal *Thinking Skills and Creativity* significantly contribute to the development of CT skills in ELT. One of the globally cited documents from *Thinking Skills and Creativity* published in 2016, titled “*Making* L*2 Learners' Reasoning Skills Visible*: *The Potential of Computer* Supported *Collaborative Learning Environments*” highlighted how multimodality fostered CT skills. The study employed an application known as ‘Digital Mysteries’ which focused on engaging students in Computer Supported Collaborative Learning Environment (CSCLE) [39]. In addition to being a highly reputed journal, *Thinking Skills and Creativity* features other internationally referenced articles on CT in ELT. Another globally cited document was ‘*An Adaptable Teacher Education Framework for Critical Thinking in Language Teaching*’ published in 2018 that shed light on the awareness among English teachers' regarding the integration of CT in ELT and the study has implemented Paul and Elder's CT framework that consists of intellectual standards, elements of thought and the intellectual virtues as the teaching pedagogy [40]. In addition, International Journal of Instruction and Theory and Practice in Language Studies were ranked the second and third most relevant sources. Similarly, Arab World English Journal and Asian EFL Journal occupy fourth and fifth positions, respectively. These journals show that CT skills and ELT have gained attention among teachers and researchers worldwide. The relevant sources attained through bibliometric analysis will enable researchers and academicians to learn more about publication trends in reputed journals.

**Table 3:** Top ten most pertinent journals.

Elements	g_index	m_index	TC	NP	PY_start
Thinking Skills and Creativity	4	0.5	61	4	2016
International Journal of Instruction	4	0.5	31	4	2018
Theory And Practice in Language Studies	4	0.2	25	9	2013
Arab World English Journal	2	0.1	12	13	2012
Asian EFL Journal	3	0.2	12	4	2014
Education Sciences	3	0.6	11	3	2021
English Teaching and Learning	2	0.2	10	2	2015
Gema Online Journal of Language Studies	2	0.3	6	2	2018
International Journal of Applied Linguistics and English Literature	2	0.2	7	2	2014
International Journal of English Linguistics	3	0.3	18	3	2018

### 4.3 Average citation counts for research on CT in ELT

**Fig. 3:**
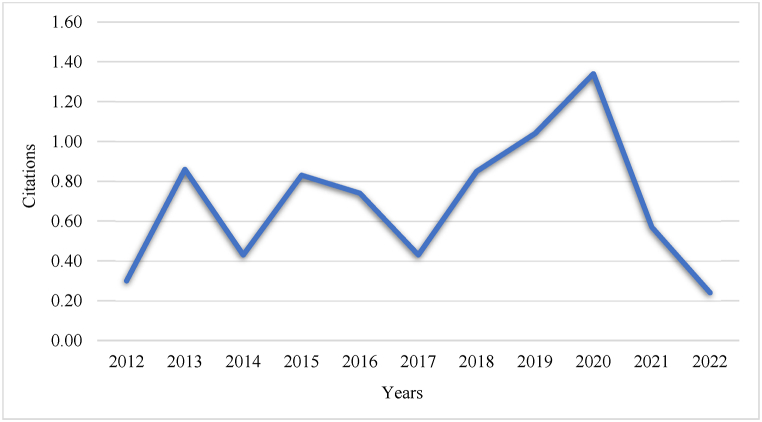
Average article citations per year.

The citation metrics provide valuable insights into the impact and influence of the prevalent research publications in this field. Citations reflect the quality and importance of the research works, as well as the frequency with which these works are referenced by other researchers. As shown in Fig. 3 a higher average number of citations per year indicates that the articles in this domain are meeting rigorous academic standards and making significant contributions.

This indicates a decline in citations in 2022 despite the increasing trend of articles on CT skills in ELT, as evidenced by the annual scientific article production in Fig. 3. In terms of annual citations, research on CT in ELT exhibits extreme volatility. The lowest annual mean total citation is 0.24 in 2022, which contradicts the annual scientific publication of articles in the same year as the production of articles is higher, but the total number of citations remains low. Meanwhile, there was a steady increase in the average number of citations of papers from 2017 to 2020. The highest mean total citation from 2012 to 2022 was 1.34, which was found to be the citation record for 2020. The most frequently cited document globally, with a total of 110 citations, was published in 2020 under the title *'Effects of Peer Assessment within the Context of Spherical Video-Based Virtual Reality on EFL Students' English-Speaking Performance and Learning Perceptions.'* In this research, a peer assessment method was implemented, placing students in a spherical video-based virtual reality setting. This approach allowed participants to engage in conversations with individuals from other countries, thereby enhancing their speaking skills. They concluded that this strategy had a positive effect on learners’ speaking skills, motivation, and CT competencies [41]. Therefore, it can be deduced that a large number of articles on developing CT in ELT with the highest global citations were published in 2020.

### 4.4 Globally cited documents

The top ten most globally cited research publications in the field of CT in ELT provide a critical perspective into the intellectual sphere of this domain. The highly impactful publications, spanning from 2012 to 2022, have garnered substantial global attention. This reflects the influence of CT related research within the discipline of ELT. The top-cited publications are presented in Table 4, which offers deeper insights into the methodological approaches and theoretical frameworks that have driven the enhancement of CT skills in SLA.

**Table 4:** Top ten most globally cited documents in the field of CT in ELT.

Authors & Journals	DOI	Total Citations (TC)	TC per Year	Normalized TC
Chein Sy, 2020, Comput Educ	10.1016/j.compedu.2019.103751	110	27.50	20.47
Yang Ytc, 2013, Comput Educ	10.1016/j.compedu.2012.12.012	49	4.45	5.20
El Soufi N, 2019, Stud Educ Eval	10.1016/j.stueduc.2018.12.006	32	6.40	6.14
Yang Ytc, 2014, Br J Educ Technol	10.1111/bjet.12080	32	3.20	7.50
Hand B, 2018, Sci Educ	10.1002/sce.21341	31	5.17	6.09
Lin M, 2016, Think Skills Creat	10.1016/j.tsc.2016.06.004	29	3.63	4.94
Eftekhari M, 2016, Educ Technol Res Dev	10.1007/s11423-016-9431-z	25	3.13	4.26
Petek E, 2018, Think Skills Creat	10.1016/j.tsc.2018.02.008	18	3.00	3.54
Alkharusi Ha, 2019, Int J Instr	10.29333/iji.2019.12231a	15	3.00	2.88
Chen J, 2018, Int J Engl Linguist	10.5539/ijel.v8n6p12	15	2.50	2.95

The article *‘Effects of Peer Assessment within the Context of Spherical Video-Based Virtual Reality on EFL Students’ English-Speaking Performance and Learning Perceptions'* published in *Computer & Education* is considered to be the most eminent research in the topic. The second most globally cited document was published by *Computer & Education*, entitled ‘*A Blended Learning Environment for Individualized English Listening and Speaking Integrating Critical Thinking’* highlight the fact that crucial 21st-century skills include CT and English communication. This study emphasized incorporating CT into learners' English listening and speaking instructions through individualized strategies, such as proficiency level grouping and individualized feedback. The results showed that students demonstrated enhanced CT and English language communication, which equips them with knowledge of 21st-century competencies [42]. The following globally cited documents were published by *Studies in Educational Evaluation* and *the British Journal of Education Technology*, as they are considered to be the leading journals with high global citations in the field of CT research in ELT. The fourth and fifth places were held by *Science Education* and *Thinking Skills and Creativity*, respectively. The global citation count of an article signifies the number of times it has been cited, demonstrating its significance to the scientific community in a specific research area.

### 4.5 The local impact of authors based on total citation counts

The local impact of authors is a key indicator of their scholarly influence in the scientific community. The current study examines the scholarly influence of 562 authors in the field based on the total citation counts of their research publications. This cumulative citation data reveals the significance of the authors and the citation metrics yield information regarding the impact of prolific researchers who are shaping the discourse of CT in SLA.

**Fig. 4:**
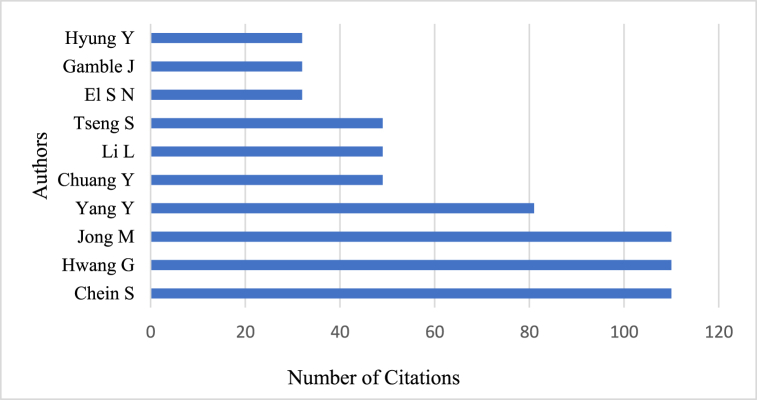
The local impact of top ten authors.

**Fig. 5:**
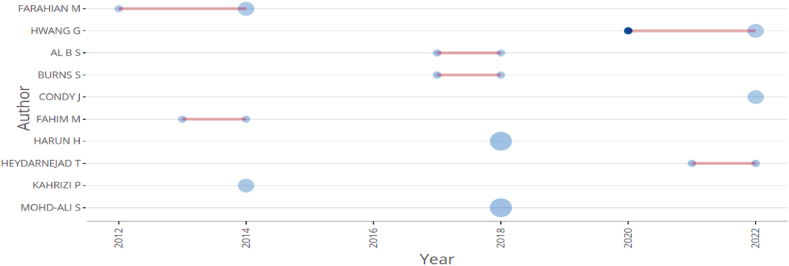
The productivity of top ten authors over time.

Fig. 4 represents the top 10 prolific authors whose works are considered influential and prominent in the field of CT research in ELT. Among the top 10 authors, Chein has the highest total citation count of 110 and possesses the globally cited document. Following closely behind are Hwan and Jong, who have the same number of total citations per year as Chein, as they co-authored the same work entitled *“Effects of peer assessment within the context of spherical video-based virtual reality on EFL students’ English-Speaking performance and learning perceptions*.” Thus, collaboration between influential authors results in high-quality publications. Furthermore, the total number of authors contributing to single-author documents is 56. Fig. 4 illustrates some of the most prolific authors and their productivity in the field of CT in ELT from 2012 to 2022. It also represents the authors' scientific output over time and displays the number of citations they possess. Fig. 5 shows authors who have a global and local impact. The lines on the figure represent the period of the authors' productivity, while the colour of the bubbles indicate the intensity of citations, respectively. In addition, the magnitude of the bubbles represents the number of documents produced over time. Thus, the bibliometric analysis examined the production of these authors from 2012 to 2022, providing insight into the extent of research produced during that time period.

### 4.6 Most relevant affiliations

The publication of research articles by authors from relevant academic and research affiliations underscores the vitality of CT in ELT. This scholarly productivity across a range of affiliated entities signifies the sustained commitment of the research community in advancing the literature in this domain. Besides, the diversity of relevant institutional affiliations contributing to this field denotes the widespread recognition and importance of CT as a crucial aspect of SLA.

**Fig. 6:**
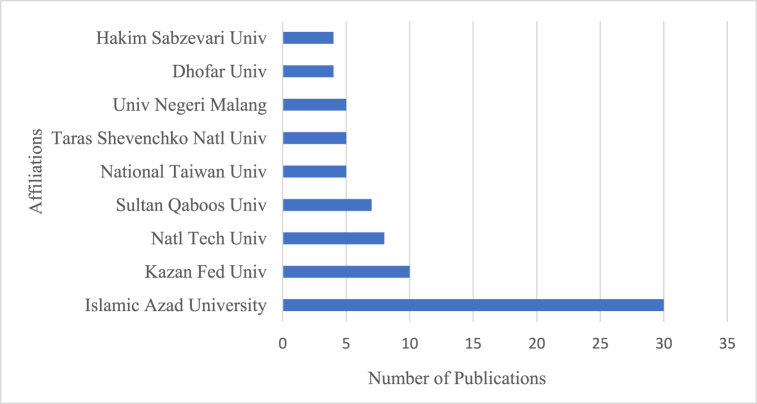
Top ten most relevant affiliations.

Fig. 6 depicts the top ten affiliations pertinent to CT research in ELT. *Islamic Azad University* published 30 articles and ranked first as the most prestigious affiliation. It published a large number of articles in 2020, the most productive year, with an intense citation period in the field of CT in ELT. The publications by *The Islamic Azad University* in 2020 includes an article titled *‘Neuro-Linguistic Programming and Its Implications for English Language Learners and Teachers*’ employed Neuro-Linguistic Programming (NLP) as a high-potential technique to develop teachers' CT skills, self-efficacy, and rapport with learners, resulting in positive effects on their academic excellence. It was suggested that supplementary practices, namely rapport, outcome thinking, sensory awareness, and behavioural flexibility, stimulate CT skills among English language learners and teachers [43]. Another article entitled ‘*Synthesizing Transformative Education with Dynamic Assessment in Developing EFL Learners’ Productive Skills'* investigated how Dynamic Assessment (DA) fostered learners' speaking and writing skills and this study focused on involving CT activities and class projects to promote learners' productive skills [44]. *Kazan Federal University* and *the National Technical University of Ukraine* are the second and third most pertinent affiliations, respectively. Therefore, the substantial number of articles produced by these affiliations indicates that the area of CT in ELT has generated interest from various countries, such as Russia, Ukraine, and the Middle East, with the top three affiliations situated in these respective countries.

### 4.7 Leading countries in the field of CT research in ELT

**Fig. 7:**
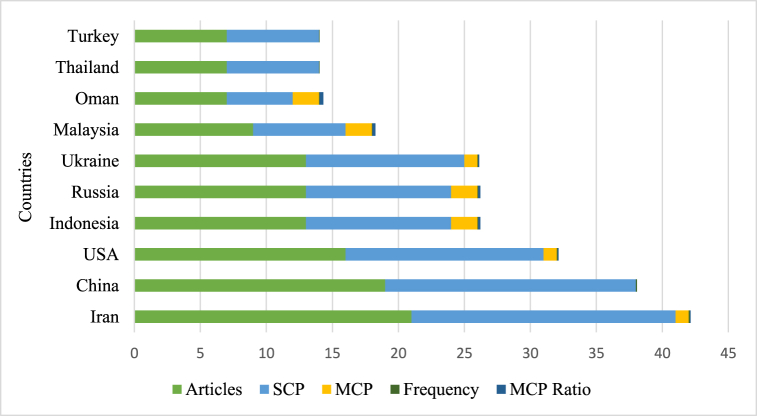
Top ten most highly relevant countries.

The geographic distribution of countries engaged in CT research offers a comprehensive understanding of the worldwide influence of this field. The patterns of this analysis suggest areas that may necessitate further exploration. The top ten relevant countries that have been highly active and influential contributors to this field are presented in Fig. 7 offering a visual depiction of the worldwide impact and relevance of this research area.

It includes Single Country Publication (SCP), Multiple Country Publication (MCP), the frequency of the countries, the ratio of MCP, and articles published in the countries. It is evident that SCP is higher than MCP, and the collaboration between countries is illustrated by the MCP Ratio. Oman has the highest Multiple Country Publication (MCP) ratio at 0.286, while China, Thailand, and Turkey have less MCP. However, China's productivity is high, indicating that the majority of research is conducted within the country, for instance, a study titled *‘Effects of peer assessment within the context of spherical video-based virtual reality on EFL students’ English-Speaking performance and learning perceptions'* was co-authored by Chein, Hwang, and Jong who are all from China. Researchers in China tend to collaborate with domestic authors rather than collaborating with international authors. Iran tops the list among the most relevant countries of authors in the field of CT research in ELT. Iran exemplifies the observation that most collaborations occur within the country and how researchers seem more productive within the country. In 2021, an article titled ‘*The Relationship between Critical Thinking, Self-regulation, and Teaching Style Preferences among EFL Teachers: A Path Analysis Approach’* was co-authored by Heydarnejad, Fatemi and Ghonsooly, all of whom were from Iran [45]. It can be inferred that Iran is a significant contributor in this field. Additionally, other countries such as China, the United States, Indonesia, and Russia are considered relevant in this field.

**Fig. 8:**
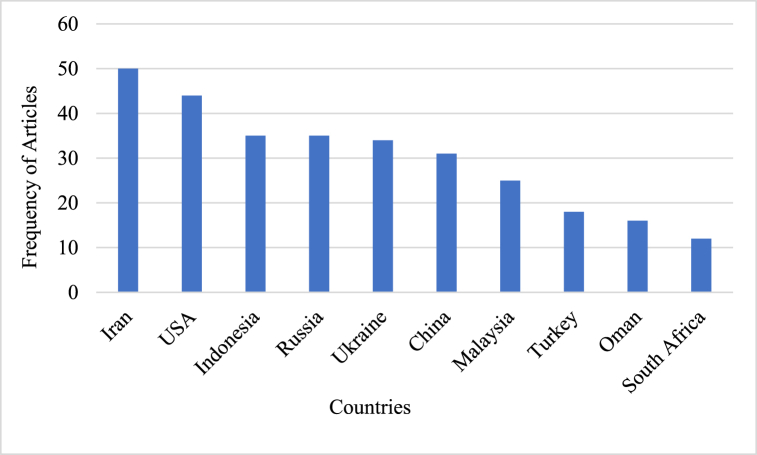
Distribution of the top 10 countries with the most publications in CT.

Leading nations with high productivity frequencies are shown in Fig. 8. The top five positions are Iran, the United States of America (USA), Indonesia, Russia, and Ukraine, with the highest frequency of article production. Although China is the most cited country, the number of articles published is less than that of Iran. Countries like Turkey, Oman, and South Africa ought to be acknowledged for their contributions to the field of CT research, as Oman, which has the least frequency among all countries, claims the highest Multiple Country Publication (MCP). Developing research on CT in ELT from 2012 to 2022 in nations with minimal output is essential for stimulating their future investigative pursuits in this field.

### 4.8 Most cited countries

**Fig. 9:**
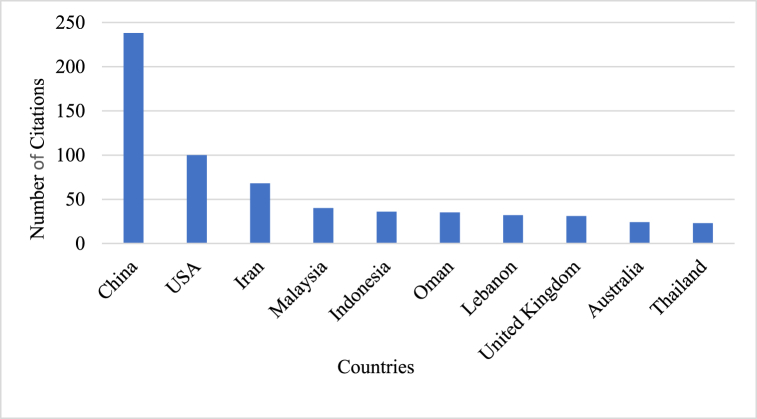
The top 10 countries with high citations in the field of CT in ELT.

The most cited countries in the domain of CT in ELT have been identified by analyzing the citation metrics of research articles published by authors affiliated with these nations. As shown in Fig. 9. China emerges as the leading contributor in terms of citations, with 238 citations attributed to research articles from this country. The dominance of China in the citation rankings underscores its leading role and influential contributions to this field.

The efficacy of China's productivity in publishing articles in this specific field is demonstrated by the fact that the document with the highest number of global citations, titled *‘Effects of Peer Assessment within the Context of Spherical Video-Based Virtual Reality on EFL Students’ English-Speaking Performance and Learning Perceptions,* was published by Chinese authors [41]. The United States stands in second place with a total citation count of 100, which is low compared to China but high compared to other countries. Iran holds third place with a total citation of 68, whereas Malaysia and Indonesia occupy fourth and fifth places, respectively. There are several countries with fewer than 50 citations, suggesting that there is potential for enhancing the efficiency of studies related to CT in ELT. Compared to other countries, Oman has a relatively low level of productivity, but it possesses high MCP ratio than any high-frequency countries. Therefore, the intensity of CT research regarding ELT is expected to increase in the near future in these countries.

### 4.9 Research trends in CT and ELT

#### 4.9.1 Treemap visualization of high-frequency words

**Fig. 10:**
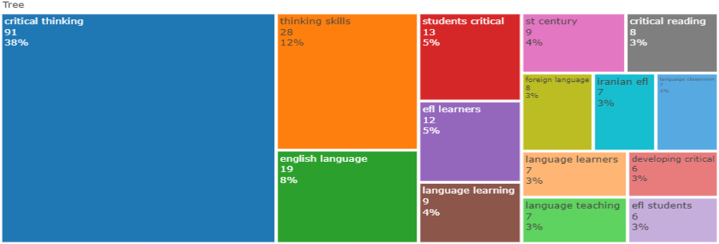
The most relevant keywords.

**Fig. 11:**
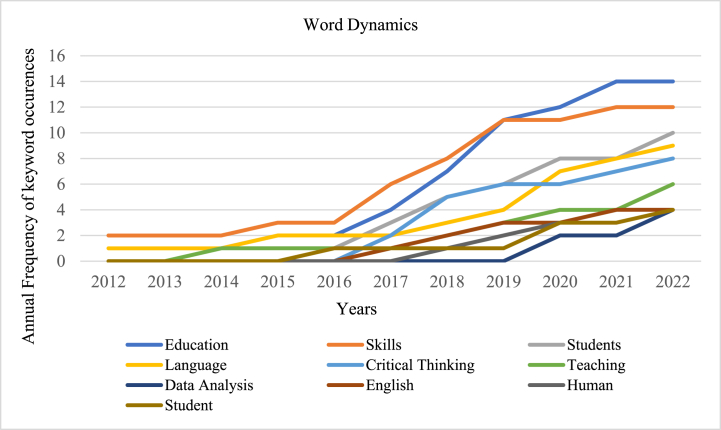
The top 10 high-frequency keywords per year.

The current study presents a tree map of high-frequency words to decipher the research conducted in this area and predict future research aspects using frequently occurring terms. Thus, the tree map assists in interpreting the trends and topics in this field of research. It depicts concepts that have drawn attention in the field of CT research in ELT. Tree-map analysis was carried out using the authors' keywords, and bigram frequency was used to measure the frequency of the words. The larger the space occupied by the keyword in the tree map, the higher is the frequency of the word. In Fig. 10 *critical thinking* is the keyword used persistently with a frequency of 38 %, followed by *thinking skills* with a frequency of 12 %. The keyword *English language* was also used in the title recurrently, with an 8 % frequency rate. Keywords such as *EFL learners, language learning,‘st century, foreign language, language learners, language teaching, critical reading, Iranian EFL, language classrooms* occur frequently. Fig. 11 illustrates the annual frequency of the top ten keywords, demonstrating their growth over the past decade. The five high-frequency keywords that occur per year are *education, skills, students, language, and critical thinking,* which determine the direction for future research in a specific field. The word dynamics itself indicate the need to integrate CT skills into ELT.

#### 4.9.2 Network visualization of high-frequency words

**Fig. 12:**
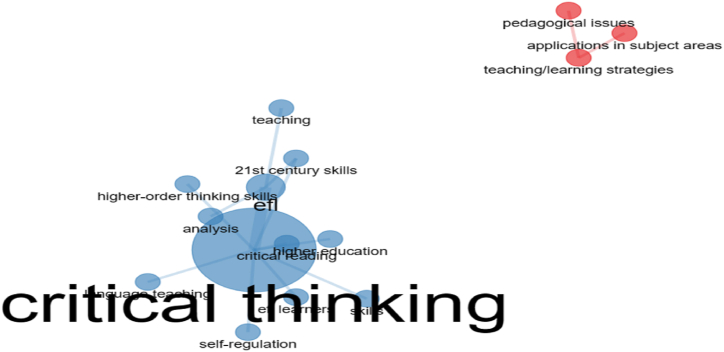
Network of keyword co-occurrence.

The co-occurrence of keywords is a hotspot for future research. Fig. 12 shows the network of keywords that occur repeatedly in CT research. The keywords recurred at least twice in the same study, indicates exponential growth of that specific field. In addition, keyword co-occurrences obtained through the network approach reflect the frequency of the words employed in a particular study. In addition, Fig. 12 illustrates the trending topics of CT in ELT through networks, where every node in the network corresponds to a keyword. The significance of a keyword is proportional to its size; the greater the node, the higher the frequency of the keywords. It also shows two distinct coloured nodes, where the blue node is interconnected and the keywords associated with each other are likely to become a significant research focus in the near future. Keywords of the same colour are similar in nature and the keywords in the blue node highlight that more research could happen in areas of *higher-order thinking*, *critical reading, higher education, self-regulation*, and more emphasis will be placed on research focusing on 21st*-century skills*. It is found that the keywords mentioned above are closely related and they occur together in studies on CT in ELT, for instance, the words s*elf-regulation and critical thinking* exist together in a study titled ‘*The Relationship between Critical Thinking, Self-regulation and Teaching Style Preferences among EFL Teachers: A Path Analysis Approach’* revealed that self-regulatory practices among teachers enhance the quality of instruction and make the classroom student-centered [45]. Likewise, the keyword occurrences in the blue node emphasize the incorporation of self-regulatory strategies such as goal setting, self-supporting thoughts, and self-evaluation, which aid in comprehending the concepts of a particular field. The red node represents keywords that highlight another research hotspot, including *pedagogical issues, teaching/learning strategies, and practical applications in the subject areas*. Therefore, future research should focus on proposing appropriate teaching strategies to address the pedagogical issues related to CT in ELT.

### 4.10 Evolution of themes

The evolution of research themes in this field can be traced through the thematic transition of the keywords used in the previous research publications. The patterns of keywords can help future researchers understand the concepts and themes in this scholarly area. In addition, the thematic evolution of keywords in the corpus of research on CT in ELT offers a lens into the development and progression of major research areas within this field.

**Fig. 13:**
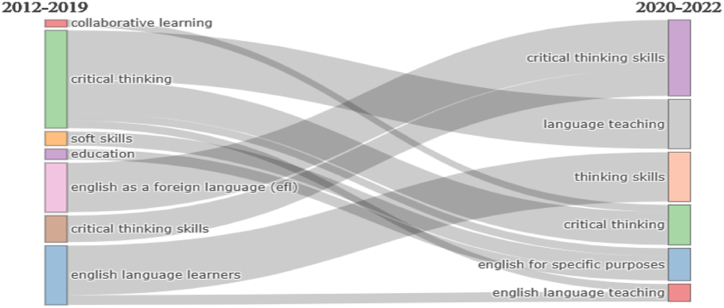
Progression of themes from 2012 to 2022.

Fig. 13 demonstrates the progression of themes from 2012 to 2022 related to this area. This thematic evolution indicates the rapid growth of the research area from 2012 to 2022. It is divided into two phases, 2012–2019 and 2020–2022, representing that CT research in ELT has recently undergone robust evolution as it is highly pertinent to the current era. The conversion correlation of themes represents the significance of language teaching in the second phase (2020–2022). As shown in Fig. 13. CT skills were considered important during the second phase. The deeper the line, the greater the prominence of the subject throughout the stage. Themes such as English for Specific Purposes (ESP) and ELT have gained prominence in the second phase. These thematic evolutionary pathways indicate future research directions and emerging topics in CT. Thus, ELT can be viewed as a dynamic field that has undergone significant change over the years.

## 5 Discussion

In light of the comprehensive bibliometric analysis conducted, this section delineates the salient findings of this investigation. The primary objective of RQ1 is to evaluate research trends in the field of CT in ELT. Through an analysis of publication and citation metrics, it is feasible to ascertain the future trajectory of the research domain. The publication trends in Fig. 2 depict gradual growth in terms of the production of articles. The key outcomes of RQ1 indicate that the yearly scientific output and growth rate of articles in this area are both substantial and steady. It is evident that there was a sudden increase in publications in 2018, which shows the importance of CT in ELT. A notable discovery was that the highest citation rate in the year 2020 was 1.34. The results of this review align with the findings of other significant bibliometric studies, including a bibliometric analysis of CT for learners of higher education, which indicates a substantial increase in articles from 2017 to 2021, thereby demonstrating the growing interest of researchers in CT. A previous investigation on the trends of CT displayed a parallel pattern, demonstrating a surge in citations over the past two decades, corresponding to the years of greatest scientific production [46,47]. Thus, the publication and citation records provide an overview of this field to researchers and academicians.

The second research question aims to identify important journals, authors, and their works. This analysis provides valuable insights into the key contributors to the field. The present study determined *Thinking Skills and Creativity* journal as the prominent journal in the field of CT in ELT through the analysis. Prior bibliometric study on the evolution of CT in primary education also identified *Thinking Skills and Creativity* as key contributor to the field of CT. Therefore, this study produced results consistent with those of previous studies in the same field [48,49]. The overall analysis of RQ2 provides insights into the most influential authors, documents, and sources, which may guide researchers in this field.

The RQ3 examines the institutional affiliations and countries involved in the research, revealing that China is the most frequently cited country in this field, followed by the United States. In contrast, a previous study that conducted a bibliometric analysis of articles on developing CT skills in early childhood found that publication frequency was higher in the United States and Germany. In addition, a recent bibliometric study on CT in education analysed all the prior research conducted between 1980 and 2022 and found that the most cited countries were the USA and Australia [50,51]. Thus, previous studies that performed bibliometric analyses in different timelines and diverse fields have led to a difference in some of the results of the present study. Furthermore, the current study found that *Islamic Azad University* played a crucial role in the field of CT by publishing a large number of research articles on CT. Similarly, a bibliometric study disclosed that *The Islamic Azad University* in Iran emerged as the most productive institution in the field of CT in ELT [52]. The comprehensive examination of RQ3 offers valuable perspectives on the most significant countries and institutions, enabling researchers to follow the research trajectory.

The objective of RQ4 is to analyse the current trends, prominent keywords, keyword co-occurrences and progression of themes in this field. The Word dynamics (Fig. 11.) is obtained through a network graph to showcase the top five most common keywords for each year. They are *education, skills, students, language, and critical thinking* which determine the direction for future research in this specific field. The analysis of high frequency words (Fig. 13.) include co-occurrence of keywords such as *critical thinking, critical reading, language teaching, higher-order thinking skills, and self-regulation*. The other node in Fig. 13 denote keywords such as *pedagogical issues, application in subject areas, and teaching/learning strategies.* Likewise, a bibliometric study on learners' CT skills depicted a network visualization of authors’ keywords such as *critical thinking, high school student, and evaluation* [53]. The co-occurrence of keywords, such as *critical thinking, higher education, self-regulated learning, metacognition, and problem solving* holds significant scope for research in this area. Similarly, the analysis of the current study revealed that several key concepts are interconnected, including *critical thinking, self-regulation, self-evaluation, teaching/learning strategies, and learning models.* These findings shed light on the significance of these keywords in future research in this particular field [54]. Therefore, this review has meticulously addressed and resolved all the research questions posed for this investigation and contributed to the existing body of knowledge on CT.

## 6 Conclusion

The present study conducted a bibliometric analysis of articles from 2012 to 2022, providing a comprehensive review of CT in ELT. The annual production of articles indicates significant growth, demonstrating increased scholarly interest over the past decade. The proliferation of articles highlights the interest of researchers in this field. A detailed bibliometric analysis identified relevant journals, authors, countries, and affiliations, illustrating the research continuum in this area. The research was in its early stages from 2012 to 2018 but gained momentum in 2018, leading to a surge in article production from 2018 to 2022.

Following the proliferation of articles in this field, the current research presents relevant sources that play a significant role in this field. Globally cited documents provide an overview of high-quality research in this field. Moreover, the total number of citations indicates the relevance of the articles throughout these years. The most-cited countries and their production over time have made valuable contributions to this field. Alongside, research is also being conducted in other nations and affiliations, for instance, China is the most cited country in CT research related to ELT, despite Iran holding the first place in terms of relevance in this field. Therefore, the recognition of contributions made by all countries is imperative, given that those in the early stages also have a significant impact, for example, the Multiple Country Publication (MCP) ratio of Oman is higher than that of other developed countries, exhibiting the interest of researchers in collaborating with other countries. Thus, the present study provides insight into the current state of CT research in the field of ELT.

## 7 Future directions and implications

Critical thinking has always been a fundamental aspect of learning a second language and its inclusion is now regarded as a global necessity for equipping students with essential skills for the 21st century [4]. In technology-enhanced learning environments, CT is promoted through strategies like collaboration, interactive classroom activities, and self-reflection exercises. The research demonstrated that CT abilities were enhanced across various academic fields, emphasizing their significance as a valuable educational outcome for students of all age groups [55]. Educational platforms like *Online Test Pad and Mindmeister* are utilized to improve students' CT abilities where the COVID-19 emphasized the importance of technology-based learning tools and influenced the outcomes of the learners positively. As digital technologies transform education, teaching CT using these tools are essential. Thus, future technological advancements are likely to spur further research into CT using digital tools [56]. The network of keyword co-occurrence (Fig. 12.) indicates that future research should address pedagogical issues in CT, emphasizing appropriate teaching/learning strategies. Future research directions may include digital tools to address pedagogical challenges in developing CT skills. Furthermore, the keyword co-occurrence analysis (Fig. 12.) also revealed keywords such as *higher-order thinking skills* and *self-regulation* which indicates that research may happen on these topics, for instance, a study investigated the impact of combined automated-teacher feedback on learners’ writing self-efficacy, self-regulation, and anxiety compared to traditional teacher-only feedback. This enhanced their self-regulation, writing self-efficacy, motivation and self-efficacy in promoting strategic writing behaviours among the learners [57,58]. In addition, a relevant study gave importance to the concept of self-regulation in promoting self-directed language learning. The study explored how self-regulation influences self-directed language learning and indicated that interest in English language learning positively affected self-directed technological activities [59].

The keyword *higher-order thinking skills* significantly influences future research in this field. Educators can enhance higher-order thinking skills and effective communication in English by integrating CT activities. This study investigated various methods, benefits, and challenges of incorporating CT into language instruction [60]. Based on the co-occurrence of *critical thinking* and *higher-order thinking skills* in the same node, as shown in Fig. 12 it is anticipated that future research in these areas has high scope. A recent study examined the effect of the flipped classroom approach on learners’ self-regulated learning and higher-order thinking skills during the Covid-19 pandemic, revealed a strong correlation between cognitive self-regulated learning strategies and higher-order thinking skills. This indicates that *self-regulation* and *higher-order thinking skills* which belong to the blue node (Fig. 12.) are interconnected, suggesting that similar future investigations could provide valuable insights [61]. Integrating computational thinking into writing courses enhances higher-order thinking and writing skills. Computational thinking concepts also strengthen CT, indicating a relationship between CT and higher-order thinking skills [62]. Additionally, a recent investigation highlighted the role of metacognitive techniques in enhancing CT skills by facilitating knowledge acquisition and promoting higher-level cognitive processes. Thus, higher-order thinking skills, self-regulation, and CT are interconnected, presenting numerous opportunities for future research in these areas [63].

The network of keyword co-occurrences in Fig. 12 highlights critical reading as a pivotal node for future research on CT in ELT. This finding aligns with a systematic review of critical reading among the learners where critical reading and thinking are essential in contemporary society. As social media has become an essential part of communication, learners are exposed to various formats, including posts, blogs, and instant messaging. Consequently, strong reading skills have become crucial for understanding and interpreting the abundance of readily available information. The sheer volume of data on social media requires a critical perspective, which makes critical reading increasingly prevalent in this era. Since the most critical reading research occurs in Asia, it is poised to become a globally significant research area in the future [64].

In addition, the thematic evolution (Fig. 13.) illustrates the progression of themes that occurred over the years where in the first phase (2012–2019) *collaborative learning* and *critical thinking* were considered as important areas that enriched the learners; later, a major transition took place, and *critical thinking skills* and *language teaching* were prioritized from 2020 to 2022. Thematic evolution depicts the future direction of research on CT skills in ELT. *Language teaching* is given importance in the second phase (2020–2022) which proves that the research direction of CT skills focused on incorporating these skills into language teaching. According to the thematic map, English for Specific Purposes (ESP) has gained prominence in the second phase (2020–2022). It is essential to acknowledge that CT skills should be developed not only in academic environments but also tailored to meet the specific needs and contexts of learners. Therefore, ESP emerges as a crucial area for future research.

The results of this study indicate that it will motivate researchers to explore the gaps in CT research within the field of ELT, thus shaping future research directions. However, this review has its own limitations, since it covers the productivity of articles from 2012 to 2022, focuses only on research regarding CT skills in ELT and analyses articles only from the Scopus and Web of Science databases. Articles published in languages other than English were also excluded. It is suggested that future studies should carry out bibliometric analysis on various databases, consider articles from other analytical perspectives, and cover more years. During manual screening, some texts were overlooked because of a lack of emphasis on language and thinking skills. Future studies may address these gaps and broaden their scope to ensure a comprehensive analysis.

## CRediT authorship contribution statement

**M.P. Arthi:** Writing – original draft, Visualization, Software, Methodology, Data curation, Conceptualization. **S.N.S. Gandhimathi:** Writing – review & editing, Supervision, Methodology, Conceptualization.

## Ethics approval and consent to participate

This study did not require the approval of an ethics committee since we analysed a secondary database.

## Data availability statement

Data will be made available on request.

## Funding

This research did not receive any specific funding.

## Declaration of competing interest

The authors declare that they have no known competing financial interests or personal relationships that could have appeared to influence the work reported in this paper.
